# Interdisciplinary Surgical Approaches in Vaginal and Perineal Reconstruction of Advanced Rectal and Anal Female Cancer Patients

**DOI:** 10.3389/fonc.2020.00719

**Published:** 2020-05-13

**Authors:** Raymund E. Horch, Ingo Ludolph, Aijia Cai, Klaus Weber, Robert Grützmann, Andreas Arkudas

**Affiliations:** ^1^Department of Plastic and Hand Surgery, University Hospital Erlangen—Friedrich Alexander University of Erlangen-Nuernberg FAU, Erlangen, Germany; ^2^Department of Surgery, University Hospital Erlangen—Friedrich Alexander University of Erlangen-Nuernberg FAU, Erlangen, Germany

**Keywords:** VRAM, rectal cancer, transpelvic vertical rectus abdominis myocutaneous flap, anal cancer, vaginal reconstruction

## Abstract

Relapsing or far advanced rectal and anal cancers remain difficult to treat and require interdisciplinary approaches. Due to modern standard protocols all patients receive irradiation and neoadjuvant chemotherapy—and in case of a relapse a second irradiation—rendering the surgical site prone to surgical site infections and oftentimes long lasting sinus and septic complications after exenteration in the pelvis. Despite an improved overall survival rate in these patients the downside of radical tumor surgery in the pelvis is a major loss of quality of life, especially in women when parts of the vagina need to be resected. Derived from our experince with over 300 patients receiving pelvic and perineal reconstruciton with a transpelvic vertical rectus abdominis myocutaneous (tpVRAM) flap we studied the impact of this surgical technique on the outcomes of female patients with or without vaginal reconstruction following pelvic exenteration. We found out that the tpVRAM flap is reliably perfused and helps to reduce long term wound healing desasters in the irradiated perineal/vaginal/gluteal region.

“*Interdisciplinary surgical approaches in vaginal and perineal reconstruction of rectal and anal female cancer patients with the transpelvic VRAM flap – long term results in a large cohort.”*

## Background

Especially in female patients with advanced rectal or anal cancer the rate of perineal and pelvic wound complications after a neoadjuvant chemoradiotherapy and abdominoperineal resection (APR) has been described up to 60% of patients (1). The most frequent sequelae include acute perineal abscesses, perineal herniation and long term wound dehiscence (2–6).

The value of the transpelvic (tp) Vertical Rectus Abdominis Myocutaneous (VRAM) flap to reconstruct the perineum has been reported previously (1, 7–10). The non-irradiated myocutaneous flap can be used to cover the resected sacrum and occlude the pelvic entrance. Thus, the previously described surgical complications can be reduced by the obliteration of the dead space utilizing well-vascularized tissue while also reconstructing the perineal soft tissue and skin defect in far advanced and relapsing rectal carcinoma with abdominoperineal resection and (neo)adjuvant chemoradiotherapy. In female patients with relapsing or advanced carcinoma in the pelvis either the posterior part or sometimes also the anterior part or even most of the vagina needs to be resected for oncological reasons and to prevent therapy resistant sinuses and fistulae (11). In these patients it has been shown that primary reconstruction of the vagina—mostly the posterior vaginal wall with a tpVRAM flap can be achieved with a low complication rate and excellent long term results concerning vaginal function and stability of the reconstruction. Technical variations have been developed over the years to fit the individual reconstructive needs. The aim of this study was to retrospectively evaluate the clinical outcome of female patients receiving a perineal and/or vaginal reconstruction using a tpVRAM flap at the University Hospital Erlangen and Erlangen Cancer Center over the past 20 years.

## Study Design, Patients, and Methods

All patients with advanced rectal, anal or vulvar cancer who underwent preoperative chemoradiotherapy and abdominoperineal resection (APR) with an interdisciplinary reconstruction utilizing a pedicled myocutaneous flap during a 20-years period at the University Hospital Erlangen and Erlangen Cancer Center were retrospectively reviewed.

Between March 1st of 1999 and August 1st 2019 361 (219 male and 142 female) patients were identified, who presented with rectal, anal, or vulvar carcinoma, or locally advanced or relapsing perineal/groin malignant tumors and who were surgically treated in an interdisciplinary approach using a reconstruction with a tpVRAM flap after discussing the procedure according to the votes to the tumor board of a comprehensive cancer center.

## Results

**Figure 1:**
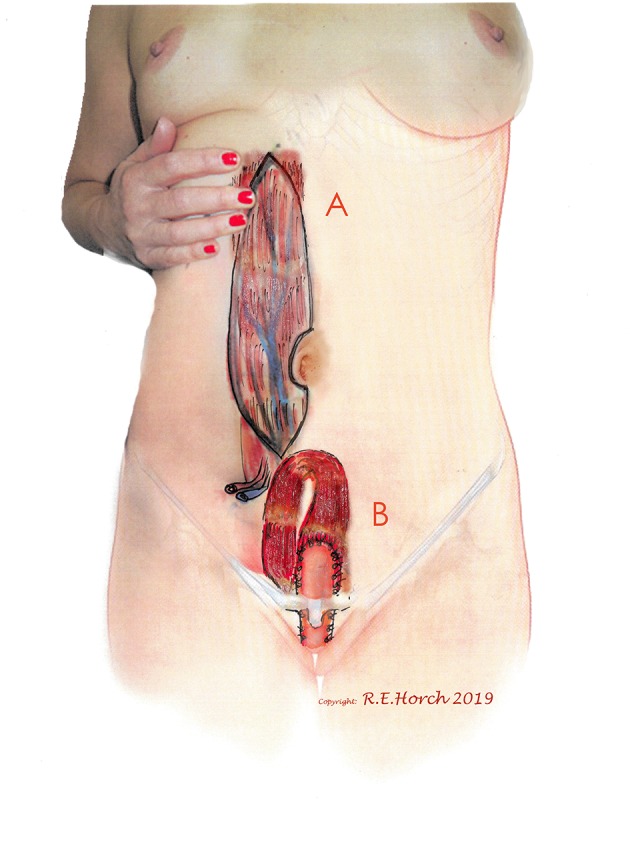
**(A)** Vertical rectus abdominis myocutaneous (VRAM) Flap preoperatively outlined on standing female patient. **(B)** Planned intrapelvic VRAM flap transposition to reconstruct the posterior vaginal wall.

Among the 142 female patients 77 female patients with vaginal wall resections were treated with a vascularly pedicled tpVRAM flap to reconstruct the vaginal wall and/or the perineum and sacrum (Figure 1).

**Figure 2:**
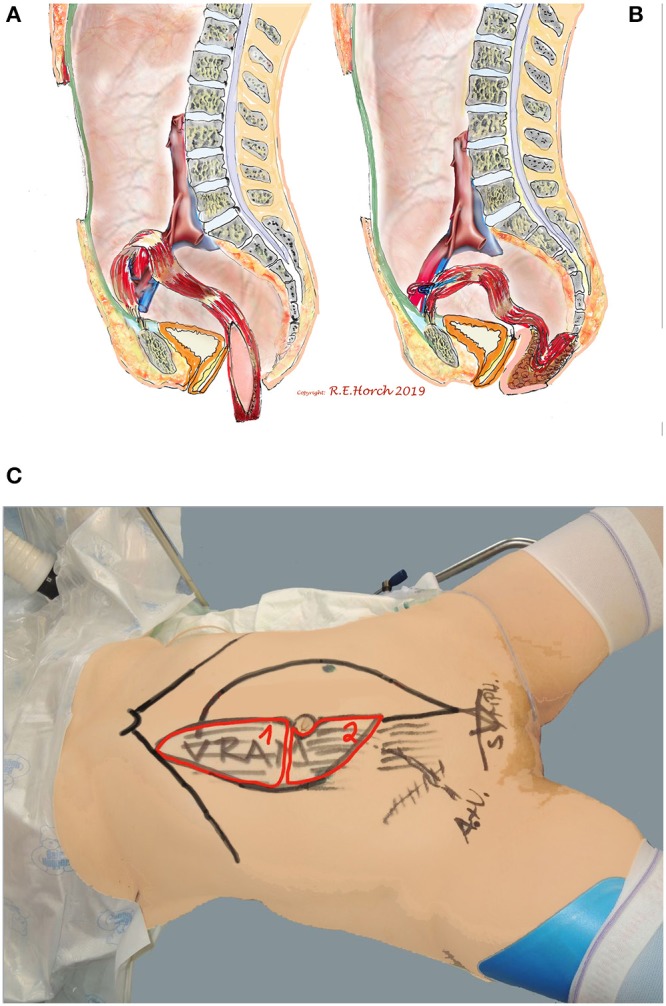
Schematic drawing of principle of vaginal wall reconstruction with pedicled transpelvic VRAM flap. **(A)** VRAM flap mobilized and routed through pelvis into resectional defect. **(B)** VRAM flap sutured to remaining anterior vaginal wall and constructing new posterior vaginal wall. **(C)** Positioning of patient on operating table and schematic drawing of planned bi-parted skin island of VRAM flap to reconstruct vaginal wall and perineum/sacrum defect with the same skin island.

77 patients received vaginal wall reconstruction with a tpVRAM together with perineal and sacral reconstruction as well as occlusion of the pelvic floor (Figure 2).

In 41 female patients a tpVRAM was applied to reconstruct the perineum and to cover the sacrum as well as to occlude the dead space in the pelvis following exenteration or radical resection without the necessity of a vaginal reconstruction.

In three patients a transpelvic rectus abdominis muscle only (RAM) flap was deemed sufficient and suitable to occlude the pelvic entrance and close the pevlic floor when no skin was resected or no skin defect resulted after radical tumor surgery.

Additionally 16 patients were treated with an extraabdominally routed (non-transpelvic) VRAM flap to reconstruct the vulva, vagina and the groin.

For various surgical reasons five female patients were not eligible for a tpVRAM flap and were therefore treated with either a single sided or double sided myocutaneous gluteus maximus myocutaneous advancement flap (*n* = 4) or in one case with a gracilis myocutaneous flap (*n* = 1).

In all cases the reconstructive goal was achieved and secondary wound treatment was only necessary in 5 of these patients (3.5%) for wound dehiscence between the flap and the irradiated gluteal tissue. Conservative treatment with wound care was sufficient to heal these minor complictions in the perineum. Only one revisional surgery was necessary in this group.

**Figure 3:**
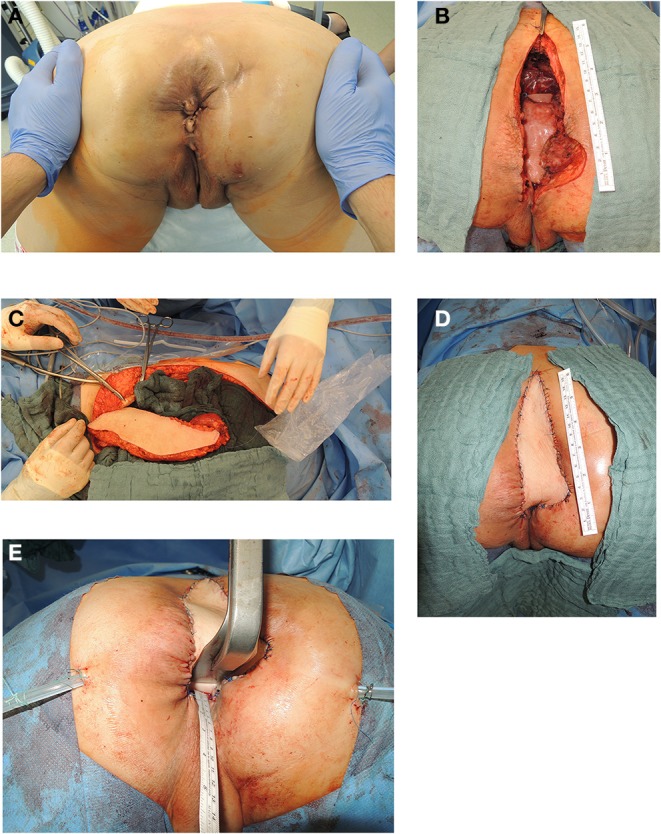
**(A)** Anatomic site of relapsing lower rectal cancer following neoadjuvant radiochemotherapy in a 55 years old female patient (patient in prone position). **(B)** Resectional defect with missing posterior vaginal wall and pelvic floor defect following radical resection of relapsing rectal cancer following cylindrical resection (patient in prone position). **(C)** Vertical recztus abdominis myocutaneous flap harvested from right abdominal wall. **(D)** Skin island of VRAM flap utilized to reconstruct posterior vaginal wall and sacral defect after coccyectomy to cover os sacrum and to close resectional skin defect (patient in prone position). **(E)** Aspect of posterior vaginal wall reconstruction and sacral defect coverage with skin island of transpelvic VRAM flap (patient in prone position).

**Figure 4:**
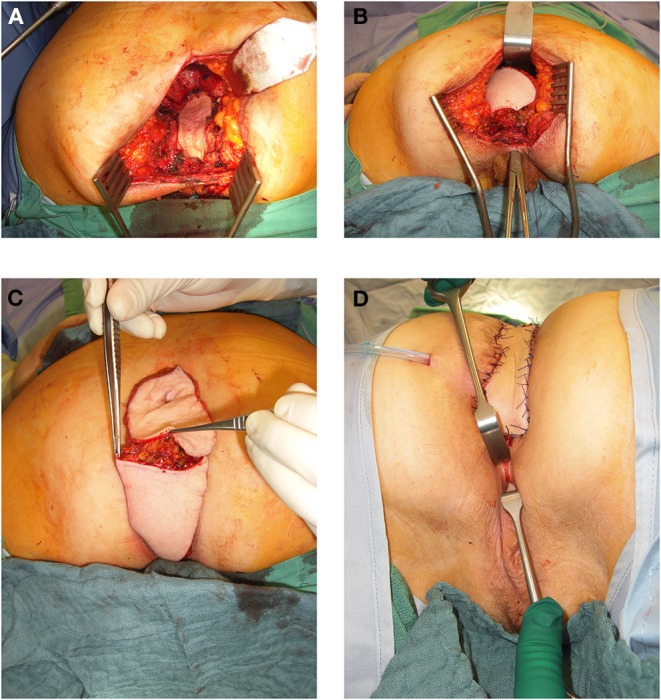
**(A)** 64 year old female patient with resectional defect after exenteration with removal of posterior vaginal wall und parts of the lateral aspects. **(B)** Transpevlic pull through of the VRAM flap into defect with patient in prone position after cylindrical excision. **(C)** VRAM flap skin island split into two parts to reconstruct the vaginal wall and covert he sacral perineal defect when vulvar entrance can be preserved. **(D)** External coverage of sacral / perineal defect with partially divided VRAM flap skin island, while second half of skin paddle was used to reconstruct the vaginal wall.

In 54% of vaginal reconstructions the skin paddle was used to simultaneously cover the perineum, sacrum and to reconstruct the posterior vaginal wall, while in 46% the skin island over the muscular flap was divided into two parts and used for vaginal repair with an appropriate amount of skin on the one hand while the second half of the island was used to cover the sacral bone following resection of the os coccygeum (Figures 3, 4).

Three patients received a RAM only to occlude the pelvic entrance, without any further complications noted postoperatively.

We observed abdominal bulging in two patients (1.4%) between 2003 and 2007. In this period we did not routinely use abdominal wall enhancement with a semisynthetic mesh inlay. These patients were treated symptomatically. After the introduction of routine abdominal wall enhancement at the muscular donor site with a semisynthetic mesh inlay we did not see any clincially symptomatic hernias or abdominal bulging during the postoperative course until now.

One obese 77 years old female patient succumbed due to pulmonary embolism 30 days after the surgical procedure with a vaginal wall and perineal VRAM flap reconstruction. There was no surgical wound healing problem with the flap or the abdominal wall.

Also in the long term no problems with sitting were noted. Since the flap has no sensitivity, we do not let the patient sit or lie in the skin island flap in order to relieve any tension for 3 weeks and then start gradual flap training with a subsequently increasing sitting time up to the 6th week postoperatively, when normal load on the flap is allowed.

Secondary thinning of a flap was necessary in one patient due to too much bulk of the skin island that did not shrink in the postoperative course and was felt to be unpleasant when sitting on the bulky mass. Surgical excision and flap thinning was performed 12 months postoperatively without complications and the further course was uneventful.

With ongoing and broadening spectrum of reconstructive challenges we learned to modify the tpVRAM flap for pelvic and vaginal reconstruction with various modifications. We proved that when the perineum or sphincter muscle can be preserved the skin paddle can be safely divided into two parts or split into y-shaped designs to exactly fit the individual reconstructive needs. This offered a greater variability of functional restoration. We also showed that the VRAM flap can be easily desepithelialized and buried under the sacral skin in case of vaginal wall resection when no extensive sacral skin resection was performed, The desepithelialized portion of the flap skin paddle served to occlude the pelvic cavity and to cover exposed bone surfaces after resection of the sacrum.

We administered betadine ointment intravaginally during the first 4 weeks and then recommended sitting baths. During the first 4–6 weeks the skin paddle of the flap within the vagina was seen to keratinize more than the rest of the non-intravaginal skin island. This phenomenon subsided in all patients after this time period and no further particularities were reported.

## Discussion

Reconstruction of the vagina after APR with or without pelvic exenteration has been an integral part of our interdisciplinary treatment algorithm and is a highly reliable single stage procedure in almost all cases (9, 12, 13). Independant from the radicality of surgery it has been shown by Bregendahl and coauthors that due to the irradiation effects urinary and sexual problems are quite common in women following radical surgery for rectal cancer (14). In addition a preoperative radiotherapy interferes with several aspects of urinary and sexual functioning (14). Postoperative bowel dysfunction following APR is affiliated with urinary dysfunction and a reduction in sexual activity and desire, as well as satisfaction (14). Vulvovaginal symptoms of non-reconstructed resectional vulvar and vaginal defects have been described to discourage patients and their partners from genital contact (15). The use of a myocutaneous transpelvic rectus abdominis myocutaneous flap may therefore not only improve wound healing, especially following a previous radiotherapy but is also suitable to reconstruct the vaginal wall and restore vaginal function. It provides ample non-irradiated, well-vascularized tissue for large pelvic soft tissue defects and skin reconstruction, that can fill up the pelvic dead space in the pelvic cavity and the perineal floor as well as the vaginal defects if necessary. It also allows for the placement of the enterostomy, and provides a chance for sexual function (16). In our study patients did not report any impairment in terms of sexual function when asked although there was no standard protocol to evaluate this topic.

Therefore, immediate single stage interdisciplinary VRAM flap reconstruction after chemoradiation and APR should be strongly considered (17). Although we did not perform standardized long term vaginal inspections, and we might have missed some complications, our own long term observations over 20 years in female patients showed that although the abdominal skin of the flap island is not primarily accomodated to the moist milieu of the vagina the skin island seems to adapt to the new surrounding conditions even when the whole vagina was reconstructed with two flaps (10). We did not observe any long term problems of the flap skin surface in the vagina. Although no true prospective studies exist that compare the outcome of vaginal reconstruction vs. non-reconstruction the additional filling of the dead space in the pelvis with a tpVRAM adds additional value to reduce surgical site infections in the irradiated pelvis (18–20). Christian et al. claimed that they in 2005 reported on their belief on the hitherto largest series of 153 patients with APR and strived to analyse the complication factors (21). They observed 22 major (14 percent) and 32 minor (24 percent) wound complications which were associated with tumor size, body mass index, diabetes, while patients with anal cancer and inflammatory bowel disease were at a higher risk to develop perineal wound complications compared to those suffering from rectal cancer (21).

Compared to the problems of secondary vaginal reconstructions it seems obvious that single stage VRAM flaps should be attempted whenever possible (22). From our observations of earlier postoperative courses before the implementation of the single stage VRAM flap procedure the significantly lower complication rate indicates a primary flap reconstruction even when the skin could be closed primarily. The objections of many colorectal surgeons who are recalcitrant to use a transpelvic VRAM flap unless primary closure is unattainable are outweighed by the advantages of perineal and vaginal reconstruction (1). In the pertinent literature reports on VRAM flap outcomes it has been mentioned that the complication rate is much lower than without flap reconstruction despite the greater extent of resection in more advanced cancers in the group with VRAM reconstructions. It was also noted that VRAM flap patients with APR in anal cancer experienced less perineal herniations vs. primary closure (0 vs. 15.4%; *P* = 0.0072) (19).

This holds also true for a comparison of VRAM flaps with other pedicled local flaps—such as a thigh flap for instance—which result in a much higher complication rate (23). These data also reflect our own results that indicate that immediate VRAM flap reconstructions result in fewer major complications than local flaps in the repair of APR and pelvic exenteration defects. Chan and coworkers reported their experience with 24 VRAM reconstructions and 6 gracilis flaps compared to 21 patients who had a primary closure in an open prospective 5 years observational study. While they did not see complications following primary closure of the unirradiated perineum they reported 17% complications after radiotherapy. They showed that a closure with a flap reduced the length of stay from 20 to 15 days, although this difference was not statistically significant (2). Spasojevic et al. also investigated whether pelvic repair with a VRAM flap following APR can improve perineal wound healing when compared to a direct perineal wound closure, which they termed non-VRAM-procedure (24). They found that in the non-VRAM group at 3 months delayed wound healing with 31.5% was more frequent than in the VRAM group (10.4%; with a *p* <0.01) (24). In their cohort of the non-VRAM group, 26.9% of patients developed pelvic abscess, compared to only 10.1% in the VRAM group (*p* <0.01) (24). Touny et al. compared a group of 60 patients which were randomly assigned to a VRAM flap procedure or a primary closure. They observed that perineal wound complications occurred in 5 patients in the VRAM group (17.2%) vs. in 14 patients in the primary closure group B (46.4%) (*P* = 0.015) (25).

Hinojosa et al. published their results with APR with vertical rectus abdominis myocutaneous flap reconstructions (VRAM) after preoperative pelvic radiation in 15 patients including 5 patients who also required posterior vaginectomy with the APR (4). They concluded that APR with VRAM flap reconstruction after preoperative pelvic radiation can be performed safely with limited wound complications and no mortality (4). Campbell and Butler compared their overall complication rates with VRAM flaps in a cohort of 185 patients with a mean follow-up of 25.1 months (26). They found that in their patients with fascia-sparing VRAM flaps resulted in significantly fewer hernias (1.5 vs. 11.5%, *p* <0.01), with less dehiscence, abdominal bulge, and evisceration (26). In our experience we noticed abdominal bulging in our earlier series until 2005 in 4% of patients but did not see any abdominal hernia so far after we changed our operative abdominal wall closure to a standardized protocol using a semisynthetic mesh to augment abdominal wall stability. This compares favorably with the data of Campbell and Butler who reported a lower number of postoperative hernias with 2.6 vs. 5.5% in patients receiving donor-site mesh inlay. On the other hand we did not find more abdominal laxity/bulge as was described by others (26). A component separation as was reported by others in the literature (27) was not necessary to close the abdominal donor site in our patients. We assume that the use of a semisynthetic mesh inlay augmentation is a cornerstone of preventing abdominal wall bulging or hernia.

This study shows our clinical experience with a low complication rate using the tpVRAM flaps in female patients for vaginal and/or perineal reconstruction over 20 years in a large cohort. To the best of our knowledge this is the largest hitherto reported series of reconstructing the vaginal wall during rectal and anal cancer surgery in a one stage procedure.

The main limitation of this retrospective study is, that, except a few patients who were – for different reasons and secondary morbidities—seen at a later stage within 1–5 years following the initial surgical therapy, we were not able to investigate all of the patients clincially since our center is serving a large regional area with great distances of our patients who had been transferred from all over the country and in a number of cases from other countries. The long term oncological outcome will be subject to further studies which aim at the oncological aspects.

## Conclusion

VRAM flap reconstruction of defects following APR—with or without vaginal wall resection—is an effective technique that reduces major perineal wound complications and wound healing delay in patients undergoing APR and irradiation without increasing early abdominal wall complications. To the best of our knowledge this is the largest hitherto reported series of reconstructing the vaginal wall during rectal and anal cancer surgery in a one stage procedure. Although the abdominal skin of the flap island is not accomodated to the moist milieu of the vagina we did not observe long term problems of the skin surface in the vagina and patients report of a possible proper sexual function with the reconstructed vagina. This type of interdisciplinary reconstruction has proven to be technically feasible in almost all cases despite of previous abdominal surgical incisions. The tpVRAM flap is reliably perfused, helps to reduce long term wound healing desasters in the irradiated perineal/vaginal/gluteal region and adds enormously to the quality of life in female patients with advanced or relapsing rectal, anal or vaginal cancer.

## Data Availability Statement

The datasets generated for this study are available on request to the corresponding author.

## Ethics Statement

The studies involving human participants were reviewed and approved by ethics committee of the Friedrich-Alexander University Erlangen-Nuernberg. Written informed consent for participation was not required for this study in accordance with the national legislation and the institutional requirements. Written informed consent was obtained from the individual(s) for the publication of any potentially identifiable images or data included in this article.

## Author Contributions

RH formulated the hypothesis, performed most of the operations, collected data and interpreted the data, and wrote the first draft of the manuscript. IL, AC, KW, RG, and AA performed surgery, and contributed to the discussion and critically reviewed the manuscript.

Figures 1, 2 were drawn by RH. All authors read and approved the final manuscript.

## Conflict of Interest

The authors declare that the research was conducted in the absence of any commercial or financial relationships that could be construed as a potential conflict of interest.
